# Perforation of an Occult Carcinoma of the Prostate as a Rare Differential Diagnosis of Subcutaneous Emphysema of the Leg

**DOI:** 10.1155/2016/5430637

**Published:** 2016-08-11

**Authors:** Mirko Velickovic, Thomas Hockertz

**Affiliations:** Department of Orthopedic Surgery, Sports Traumatology and Trauma Surgery, Städtisches Klinikum Wolfenbüttel (Wolfenbüttel Municipal Hospital), Alter Weg 80, 38302 Wolfenbüttel, Germany

## Abstract

We report a case of subcutaneous emphysema caused by perforation of the rectum due to a carcinoma of the prostate. Although rare, an abdominal cause must always be considered as a rare differential diagnosis of subcutaneous emphysema. As a matter of fact adequate diagnostic with rapid treatment is essential for the outcome.

## 1. Introduction

Carcinoma of the prostate is a frequent malignancy in men. In Germany the incidence is about 25,4%. The average patient is 69 years old when getting the diagnosis. Due to determination of the PSA (prostate specific antigen) early diagnosis is possible. Subcutaneous emphysema of the lower extremities is usually caused by an infection with aerogenic bacteria's which is often seen after major traumas like open fractures. A perforation of an abdominal organ is quite rare. We report a case of a 98-year-old patient with an occult carcinoma of the prostate with perforation of the rectum and development of subcutaneous emphysema mimicking gas edema.

## 2. Case Report

**Figure 1:**
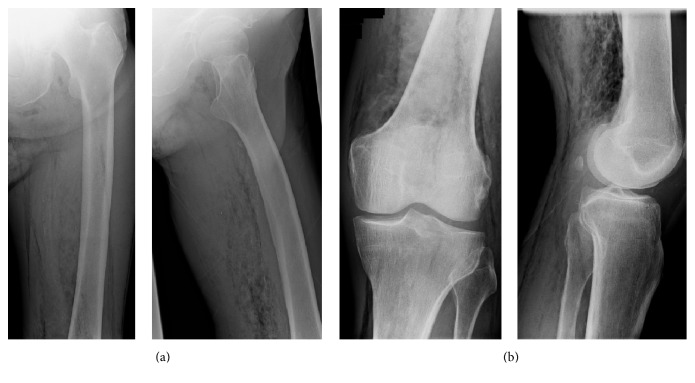
X ray of the left proximal femur and knee with demonstration of free air.

**Figure 2 fig2:**
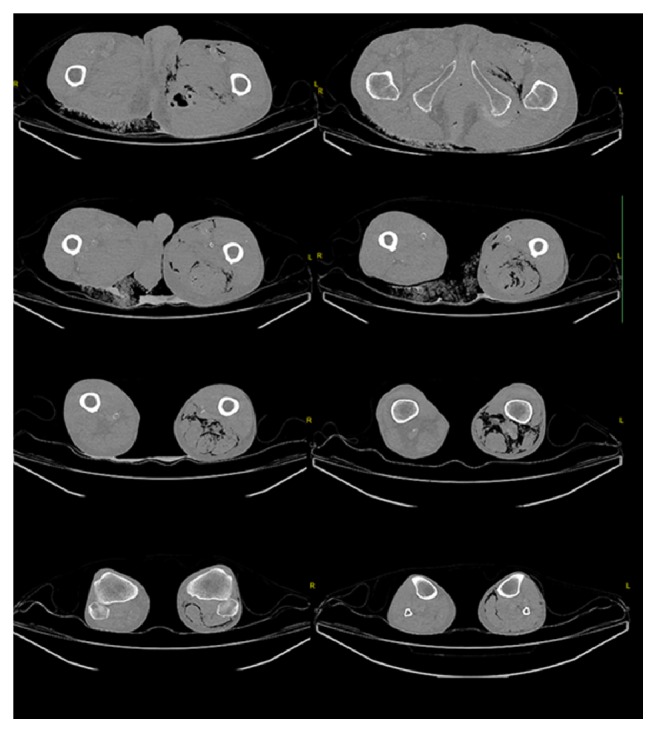
Axial CT scan of the lower leg starting from the hip to the knee with sharply demarcated free air in the soft tissue.

**Figure 3 fig3:**
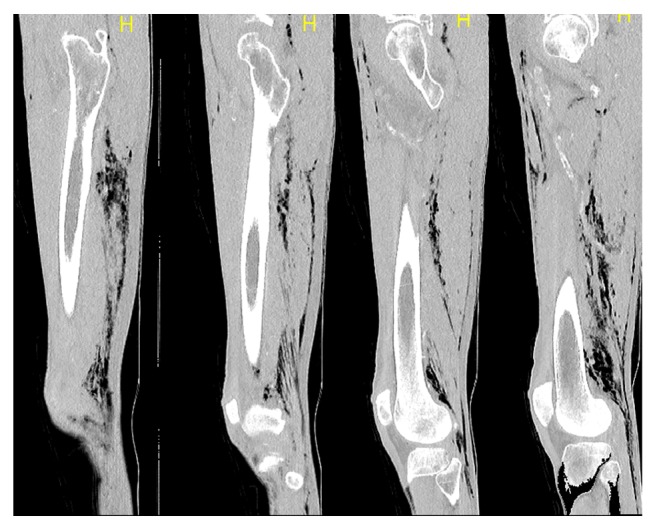
Sagittal sequence of the same CT scan.

**Figure 4:**
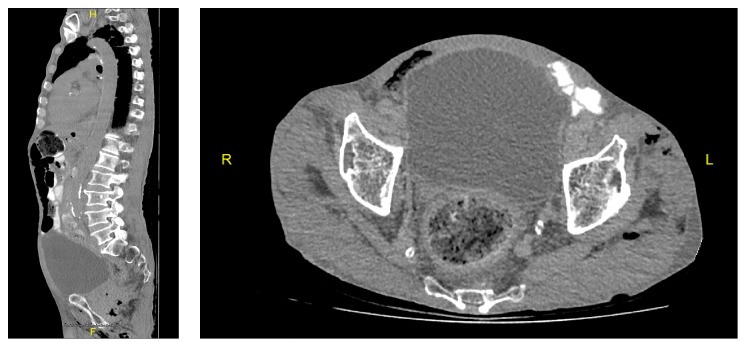
An image of tumor of the prostate.

**Figure 5:**
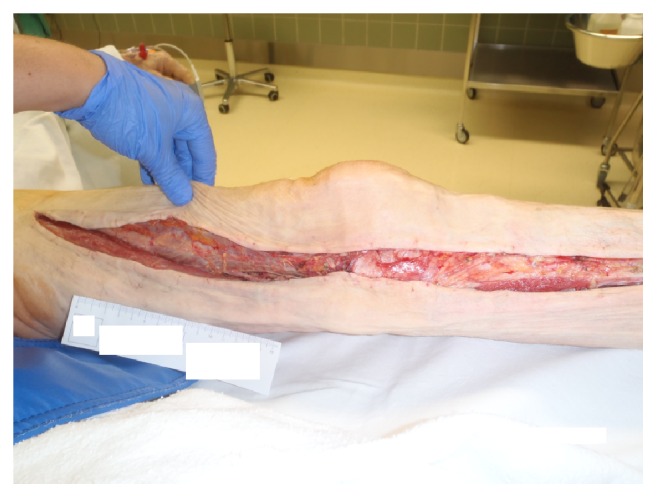
Intraoperative site with incision of the left leg.

**Figure 6:**
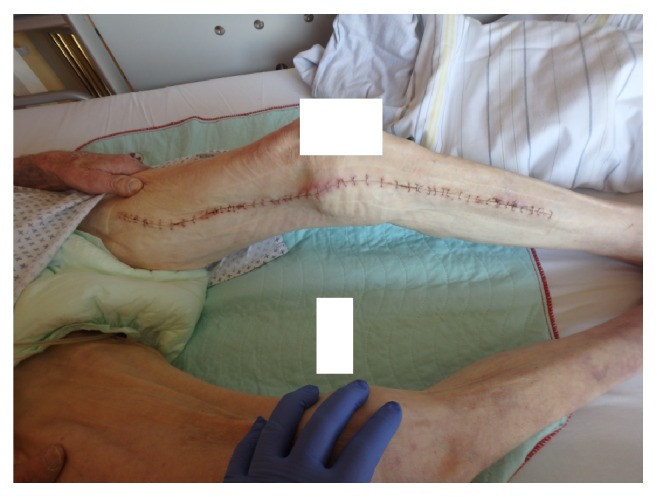
Left leg after operation with good healing process.

**Figure 7:**
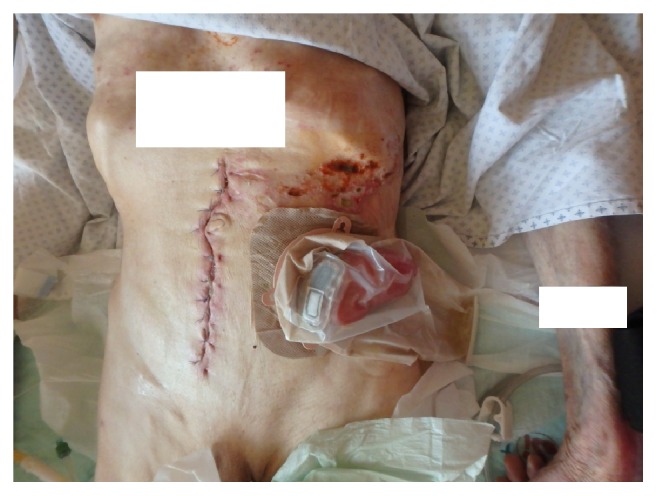
Operation site after operation with laparotomy scar and artificial anus (anus praeter).

A 98-year-old patient was admitted to the emergency department with a history of pain and swelling of the left thigh of 5-day duration after minor trauma. The patient suffered from dementia so an adequate communication was impossible. On admission, the vital parameters were normal, no fever. The physical examination revealed extensive subcutaneous crepitus in the whole left leg. There was no external wound. Other signs of inflammation were absent. Laboratory studies showed a WBC count of 11,400/*µ*L, CRP 146,2 mg/L, and procalcitonin 0,59 ng/mL. There were although signs of chronic renal failure as well as hypothyreosis. X-ray of the left leg showed massive gas shadows in the left thigh, knee, and lower leg. Furthermore there was a tenderness of the abdomen with pain in the lower abdomen during palpation. The further examination revealed a perianal abscess. We performed a CT of the abdomen/pelvis and the complete left leg. CT revealed a 9 cm large carcinoma of the prostate gland necrotizing into the rectum and into the subcutaneous tissue causing a perianal abscess formation. Additionally metastatic lesions in the right lower lung and in 4 vertebral bodies in the thoracic and lumbar spine could be found (Figures 1 [Fig fig2] [Fig fig3]–4). Contrast agent showed free air spreading from the rectum and the perforation as its origin into the gluteal region passing the adductor muscle going deep in the lower leg. An explorative laparotomy was performed. The complete exploration of the pelvic cavity was due to the extensive tumor mass not possible but an obvious perforation of the bowel was not found. There were no signs of peritonitis. The general condition of the patient was pure so we created a permanent artificial bowel outlet and collected specimen for microbiologic examination. During the digital rectal examination the tumor was clearly palpable and a biopsy sample was taken with a biopsy forceps. In a second step a fasciotomy of the left leg from the thigh to the calf was performed. Additional specimens for the microbiologic examination were collected (Figure 5). The pathologic examination of the specimen verified an adenocarcinoma of the prostate (Gleason 4 + 4). The microbiologic samples of the leg were sterile so a gas gangrene as a causative agent could be excluded. The swabs from the gut showed the presence of bacteria of the normal intestinal flora so a systemic antibiotic therapy was not necessary. Four days after the first laparotomy wound healing disturbances developed after the fasciotomy of the leg and of the abdomen so secondary wound closure was performed. During the course the patient suffered from a paralytic ileus so a third laparotomy with decompression of the small bowel and a VAC therapy was started. Finally the wound dehiscence was closed using a Vicryl mesh in a forth operation (Figures 6 and 7).

Later on the patient developed urinary tract infection which was treated with Sulfamethoxazole/Trimethoprim (Cotrim) for 19 days. Additionally the patient suffered from shingles which appeared as reddening of the skin with fluid-filled pustules on the abdomen, for which he received conservative treatment. The MRSA screening was negative. During the in-patient stay the CRP showed a declining trend with 26 mg/L; the leucocytes were already normal. Due to the advanced findings an adequate surgical treatment of the local focus was not likely to be successful anymore and prognosis becomes infaust. We started a hormone withdrawal therapy with Flutamide for 5 days as well as Enantone every 4 weeks. The patient stayed 28 days in hospital.

## 3. Discussion

**Figure 8:**
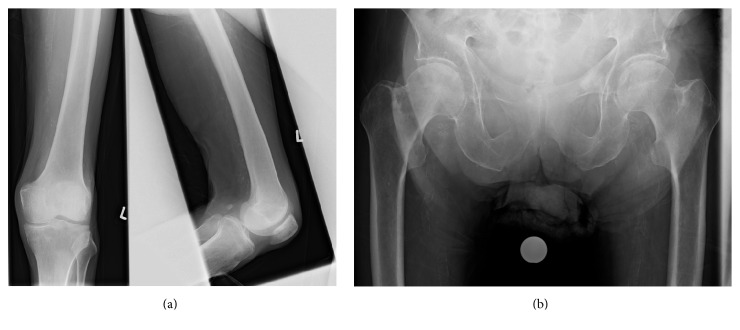
X rays of the left proximal lower leg as well of the pelvis 4 months after admission.

The presence of subcutaneous emphysema caused by the perforation of an abdominal organ is quite rare, so that misdiagnosis of this condition is presumably quite higher [1]. Nevertheless, in the presence of free air the physician should keep the possibility of an abdominal cause in mind [2]. The classic gas gangrene is an acute illness with devastating outcome. Due to the high mortality rate early diagnosis is essential [3] and requires immediate radical therapy of the soft tissue infection as well as searching of the tumor [4]. The diagnostic standards include the medical history, the physical examination, X-ray to detect free air, and laboratory investigations. The determination of the procalcitonin might be helpful. Usually the physician can find small wounds which serve as the entry portal for the organisms. In most cases the medical history is pathbreaking. What makes our case quite rare is that despite the advanced finding the patient was asymptomatic over a long period of time and the disease was unidentified. Due to the pronounced findings only a palliative care was possible. Four months after admission the patient underwent X-rays of the left leg and of the pelvis after a fall to exclude a fracture. There was no free air in the soft tissue detectible anymore (Figure 8).

## Reference Values

 CRP < 5 mg/L
 Leukocytes 4–11 × 10^3^/*μ*L
 Procalcitonin 0,05–0,5 ng/mL
 Prostate specific antigen < 4 ng/mL.
